# Global dynamic characteristics of a piecewise smooth rotor/stator rubbing system with high speed

**DOI:** 10.1371/journal.pone.0328132

**Published:** 2025-07-15

**Authors:** Shunzeng Wang, Xiaoming Liu, Yuan Liu, Jinpeng Ma

**Affiliations:** 1 School of Manufacturing Institute, Nanyang Institute of Technology, Nanyang, China; 2 State Key Laboratory of High-End Compressor and System Technology, Hefei General Machinery Research Institute, Hefei, China; 3 Key Laboratory of Computational Physics, Institute of Applied Physics and Computational Mathematics, Beijing, China; University of Botswana Faculty of Engineering and Technology, BOTSWANA

## Abstract

In this paper the global dynamic characteristics of a piecewise smooth rotor/stator rubbing system with high speed, which significantly differs from those of a low-speed system, are explored by numerical simulation and theoretical analysis. A sigmoid function is utilized to smoothen the governing equations, enabling the derivation and validation of bifurcation diagrams, as well as corresponding orbits, full spectra and Poincaré sections for both periodic and quasi-periodic motions. Additionally, the frequency relations of the quasi-periodic motions are determined. Based on the stability analysis of the periodic solutions, the presence of Hopf bifurcation boundaries, which indicate ‘jump’ phenomena between periodic and quasi-periodic motions, along with saddle-node bifurcation boundaries, is confirmed. Consequently, the global dynamic characteristics are obtained by the evolution of equilibrium solutions. Notably, zero-Hopf bifurcation is identified for the first time in the rotor/stator rubbing system with high speed. The work also reveals deep insights into the interactive effect of parameters on the dynamic characteristics of the smoothening model.

## 1 Introduction

Due to the improvement of energy efficiency of the rotating machinery, the clearance between the rotor and the stator of a rotating machine has been steeply reduced, inducing the increased risk of rotor/stator rubbing fault. During rubbing, a rotating machine performance is degraded and the catastrophic consequences of the machine may be provoked. Therefore, a large number of valuable studies on the dynamic phenomena of the rotor/stator rubbing systems surge to reveal why rubbing can happen and how rubbing should work [1–3].

The synchronous and sub-synchronous whirling motions of a horizontal Jeffcott rotor with bearing clearances are obtained by using a HB (Harmonic Balance)/AFT (Alternating Frequency/Time) technique [4]. Additionally, the nonlinear forced oscillations of a rotating shaft with the nonlinear characteristics of spring and internal damping are studied, and 1/2 order sub-harmonic oscillations of the forward and backward whirling modes are investigated [5,6]. Torsional effects in a rotor/stator contact model are discussed by numerical simulation [7,8]. From the rubbing phenomena in the rotor/stator rubbing model, it is found a rotor can remain rubbing with the stator under certain conditions, even if the initial perturbation no longer exists [9]. For the rich dynamic responses of the rotor/stator rubbing system [10–12], the studies of the dynamic behaviors and bifurcations have been drawing attention. Based on the mathematical model in two dimensions [13] and three dimensions [14], the dynamic behaviors and bifurcations are investigated by taking gravity effect into account. Correspondingly, the nonlinear dynamic characteristics of a vertical Jeffcott rotor with radial rubbing are studied without taking gravity into account [8]. In addition, the stability analysis of sliding whirl in a nonlinear Jeffcott rotor/stator system is presented by discussing the dynamic behaviors and bifurcations of the subsystems [15,16]. Then, the onset and existence conditions of dry friction backward whirl are investigated in a Jeffcott rotor/stator system [17,18]. Compared with the traditionally local analysis, the methods of global analysis can be introduced to explore the global response characteristics of the rotor/stator rubbing system. So the global response characteristics and research techniques of the rotor/stator rubbing system are determined and raised, and the five types of the co-existence of the different rotor responses are confirmed [19]. The nonlinear normal modes with a constraint condition are analytically derived from the free vibration equation of the non-conservative nonlinear subsystems of the piecewise smooth rotor/stator rubbing system [20]. Moreover, a harmonic balance method (HBM) coupled with a pseudo arc-length continuation algorithm is developed and used for the prediction of the stable dynamic behaviors of the rotor/stator rubbing system [21,22]. In addition, the dynamic characteristics of the rotor/stator rubbing system have been widely verified by experiments [23–28].

For the intrinsic discontinuity in the rotor/stator rubbing system, the non-smooth characteristics should be explicitly explained even though the bifurcation concepts and mathematical techniques of the discontinuous dynamical systems are completely undeveloped. According to the degree of discontinuity, non-smooth dynamic systems can be divided into three types, i.e., non-smooth continuous systems with the discontinuous Jacobian matrix, discontinuous systems of Filippov-type and impulse-type systems [29,30]. In the discontinuous systems, the dynamics and bifurcations of the stick-slip oscillations are developed [30–32], wherein three kinds of different friction models are considered in the rotor/stator rubbing system [30]. From the bifurcation viewpoints of non-smooth systems, the bifurcation behaviors of the non-smooth systems are explored by the generalized Jacobian matrix and fundamental solution matrix [33–35]. On the other hand, the majority of the concepts and definitions of bifurcation are also given in the piecewise-smooth systems, including border-collision bifurcation, boundary equilibrium bifurcation, limit cycle bifurcation, sliding bifurcation and grazing bifurcation, etc. [36,37]. Furthermore, these concepts can be applied in many different areas and utilized to explain the dynamic phenomena in the piecewise smooth rotor/stator rubbing systems.

The purpose of this contribution is to analyze the nonlinear dynamic behaviors and bifurcations of a Jeffcott rotor/stator rubbing system with high speed. From the numerical solutions of the response characteristics of the high-speed rubbing rotors, it can be concluded that the rotor undergoes a route from the period to the quasi-period, and then from the quasi-period to the period when the rotating speed rises in the high-speed region [38–40]. The response characteristics of the piecewise smooth rotor/stator rubbing systems have been studied by taking two subsystems into account [41], which show the dynamic behaviors of period-one and quasi-periodic motions. In practice, it is essential to get the global characteristics of the switching phenomena between the periodic-one and quasi-periodic motions. While the local singularity caused by the discontinuity has not been discussed completely when sliding occurs on the discontinuous boundary. Hence, in order to reveal the global dynamic characteristics of the rotor/stator rubbing system, a discontinuous system is transformed into a continuous system by smoothening functions [36,42]. Based on the comparison of the bifurcation diagrams in discontinuous and continuous systems, the parameters of the approximated smoothening functions can be determined. Then through the analysis of the bifurcation points between period-one and quasi-periodic solutions, the characteristics and existence conditions of responses can be explicitly verified with the aid of the eigenvalues of the Jacobian matrix.

Until now, the comprehensive studies focus on the global response characteristics in the rotor/stator rubbing system with low speed rather than high speed. Based on a mathematical model with low speed, the global dynamic characteristics, including the response characteristics of different whirling motions and their corresponding existence conditions, are theoretically determined with the aid of the characteristics of Saddle-node bifurcation and Hopf bifurcation in [6,15,18,19]. In contrast, for the high-speed rotor/stator rubbing systems, the dynamic behaviors are primarily elucidated through numerical calculations, lacking the in-depth explanation for why and how these dynamic phenomena can occur. By analyzing each local subsystem derived from discretizing solutions in high-speed micro-rotor/stator rubbing systems of MEMS (Micro Electro Mechanical Systems), the Hopf bifurcation condition derived from stability analysis of the local subsystems is just utilized to define regions of stable rubbing motions, such as quasi-periodic ones [15]. However, as indicated in [41], it is acknowledged that global response characteristics of a holonomic system cannot be completely discerned by the discretization of local solutions in general non-smooth systems, even though some characteristics can be predicted and explained. The main goal of the global dynamic analysis in this paper is to clearly tackle these issues of the holonomic rotor/stator rubbings system without relying on discretization of solutions, offering supplementary insights into the global response characteristics of a piecewise smooth rotor/stator rubbing system with high speed.

The remainder of this paper is organized as follows: In Section 2, the model of a Jeffcott rotor/stator rubbing system is introduced with the smoothening function identified through the comparison of the bifurcation diagrams. In Section 3, the dynamic behaviors of the high-speed rotor/stator rubbing system are obtained by the orbits, full spectra, Poincaré sections and bifurcation diagrams. In Section 4, based on the analytical solutions of periodic motion, the stability analysis is carried out theoretically with the aid of the eigenvalues of the Jacobian matrix. In Section 5, the characteristics of the bifurcation solutions and responses as well as their dependence on the system parameters are shown. Finally, conclusions are given in Section 6.

## 2 Mathematical model

### 2.1 Piecewise smooth rotor/stator rubbing model

**Fig 1:**
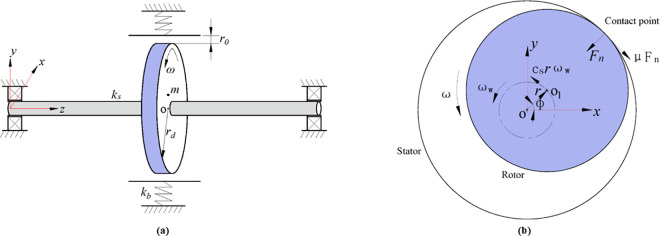
(a) Schematic diagram of Jeffcott rotor with stator. (b) Schematic diagram of the rubbing forces.

A Jeffcott rotor/stator system depicted in Fig 1 is studied in the work. The model consists of a rotor in contact with a non-rotating, compliant circular stator or a mechanical seal, as descripted in Fig 1(a). A massless and spindle shaft fixed with a disc at the middle is supported by a pair of idealized bearings. The disc with radius of $r_{d}$ is eccentric with an unbalanced mass m located at distance e from its geometrical center. The stiffness of the rotating shaft is $k_{s}$. $r_{0}$ represents the clearance between the rotor and the stator. $k_{b}$ indicates the stiffness of the annular radius spring of the stator. ω denotes the rotating speed of the rotor. During rubbing, the tangential friction force $F_{\mu }$ and the normal force $F_{n}$ are triggered at the contact points between the rotor and the stator, as illustrated in Fig 1(b). The Coulomb friction model of $F_{\mu }=\mu F_{n}$ with the dry friction coefficient of μ is employed in the system. $\omega_{w}$ denotes the whirling angular speed of the rotor. $O_{1}$ and O are respectively the geometrical centers of the rotor and the stator. ϕ is the whirling angle between the direction of the deflection of the rotor and the horizontal axis *x*. In the present analysis, the gravity is neglected.

According to the Newton’s law of motion, the governing equation of the rotor/stator rubbing system is


$$\{\begin{array}{c} {}*20lm\ddot{x}+c_{s}\dot{x}+k_{s}x+\Theta k_{b}(1-\frac{r_{0}}{r})[x-sign(v_{rel})\cdot \mu y]=me\omega^{2}\cos\omega t\\ {}[4pt]m\ddot{y}+c_{s}\dot{y}+k_{s}y+\Theta k_{b}(1-\frac{r_{0}}{r})[sign(v_{rel})\cdot \mu x+y]=me\omega^{2}\sin\omega t\\ {}[4pt]v_{rel}=r_{d}\omega +r\omega_{w} \end{array}$$ (1)

where $c_{s}$ is damping ratio of the rotor and $v_{rel}$ is the relative velocity between the rotor and the stator at the contact point with $sign(v_{rel})$ representing the direction of dry friction force. $r=\sqrt{x^{2}+y^{2}}$ is the radial displacement of the rotor. Θ is Heaviside function with Θ=0 for $r<r_{0}$ and Θ=1 for $r\geq r_{0}$.

For convenience of study, the governing equation of Eq (1) can be rewritten as the non-dimensional form.


$$\{\begin{array}{c} {}*20lX^{\prime \prime }+2\zeta X^{\prime }+\beta X+\Theta (1-\frac{R_{0}}{R})[X-sign(V_{rel})\cdot \mu Y]=\Omega^{2}\cos\Omega \tau \\ {}[4pt]Y^{\prime \prime }+2\zeta Y^{\prime }+\beta Y+\Theta (1-\frac{R_{0}}{R})[sign(V_{rel})\cdot \mu X+Y]=\Omega^{2}sin\Omega \tau \\ {}[4pt]V_{rel}=R_{d}\Omega +R\Omega_{w} \end{array}$$ (2)

where the non-dimensional variables and parameters are defined as


$$\begin{array}{c} X=\frac{x}{e},\begin{array}{c} {}*20l \end{array}Y=\frac{y}{e},\begin{array}{c} {}*20l \end{array}R=\frac{r}{e},\begin{array}{c} {}*20l \end{array}R_{0}=\frac{r_{0}}{e},\begin{array}{c} {}*20l \end{array}R_{d}=\frac{r_{d}}{e}\\ X^{\prime }=\frac{dX}{d\tau },\begin{array}{c} {}*20l \end{array}Y^{\prime }=\frac{dY}{d\tau },\begin{array}{c} {}*20l \end{array}\zeta =\frac{c_{s}}{2\sqrt{k_{b}m}},\begin{array}{c} {}*20l \end{array}\beta =\frac{k_{s}}{k_{b}}\\ \omega_{0}=\sqrt{\frac{k_{b}}{m}},\begin{array}{c} {}*20l \end{array}\tau =\omega_{0}t,\begin{array}{c} {}*20l \end{array}\Omega =\frac{\omega }{\omega_{0}},\begin{array}{c} {}*20l \end{array}\Omega_{w}=\frac{\omega_{w}}{\omega_{0}} \end{array}$$

According to Heaviside function Θ in Eq 2, the non-degenerate scalar function of $\left(R_{0}-R\right)$ on the system states *X* and *Y* is defined as the discontinuous boundary, across which the discontinuities of the piecewise smooth rotor/stator rubbing system occurs. It is noteworthy that the vector fields and their trajectories of the piecewise smooth system cannot sliding on the discontinuous boundary of $\left(R_{0}-R\right)$ [32]. This means that the deflection of rotor can only run across through the discontinuous boundary, once rubbing occurs with $R\geq R_{0}$. Thus the global dynamic characteristics induced by the discontinuous boundary of $\left(R_{0}-R\right)$ should be comprehensively studied for the piecewise smooth rotor/stator rubbing systems.

### 2.2 Rotor/stator rubbing model with smoothening function

In order to reveal the dynamics of the piecewise smooth rotor/stator rubbing system governed by Eqs (1) and (2), the smoothening function of sigmoid function sigm(R) [36] instead of the discontinuous Heaviside function Θ is introduced for numerical simulation and theoretical analysis. Then the governing equation of the smoothening rotor/stator rubbing system is shown as follows.


$$\{\begin{array}{c} {}*20lX^{\prime \prime }+2\zeta X^{\prime }+\beta X+sigm(R)(1-\frac{R_{0}}{R})[X-sign(V_{rel})\cdot \mu Y]=\Omega^{2}\cos\Omega \tau \\ {}[5pt]Y^{\prime \prime }+2\zeta Y^{\prime }+\beta Y+sigm(R)(1-\frac{R_{0}}{R})[sign(V_{rel})\cdot \mu X+Y]=\Omega^{2}sin\Omega \tau \\ {}[5pt]V_{rel}=R_{d}\Omega +R\Omega_{w}\\ {}[5pt]sigm(R)=\frac{1}{1+e^{\kappa (R_{0}-R)}} \end{array}$$ (3)

where κ is a control parameter of smoothness.

**Fig 2:**
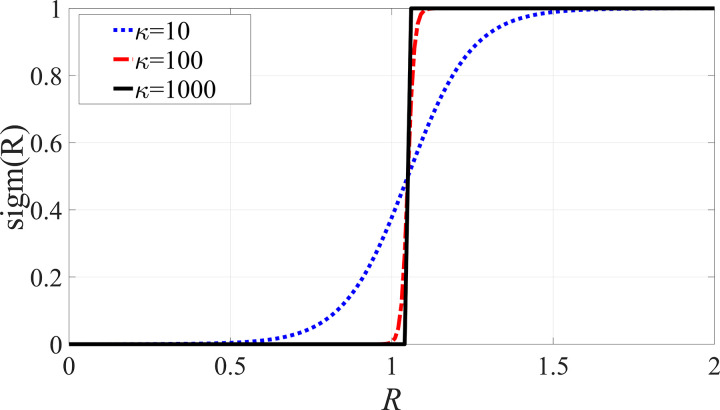
Sigmoid function sigm(R) under the different control parameter κ.

The values of sigmoid functions sigm(R) as the different values of κ are shown in Fig 2. It is seen from the lines of κ=10, κ=100 and κ=1000 that the larger the value of the control parameter κ, the closer the values of sigm(R) are to those of Heaviside function Θ. Especially, when the control parameter takes a very large value, i.e., κ=1000, the line of sigm(R) can almost overlap that of Θ. While the tradeoff is that the large value of a control parameter κ can dramatically increases the computational expense and the degree of difficulty of the global analysis. So the value of a control parameter κ is not the larger the better, but within a suitable range in the rotor/stator rubbing system. Only by comparing the dynamic behaviors of the piecewise smooth system and the smoothening system can the control parameter κ of the rotor/stator rubbing system be identified.

**Fig 3:**
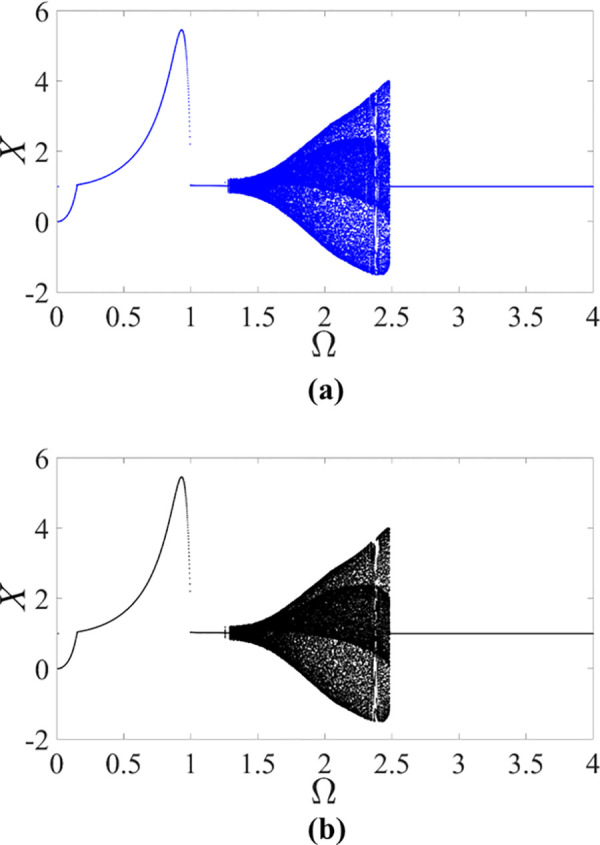
Bifurcation diagrams of the rotor/stator rubbing system with ζ=0.05, β=0.04, μ=0.08 and $R_{0}=1.05$ obtained from (a) the piecewise smooth governing equation, (b) the smoothening governing equation with κ=73.35.

Firstly, with the variation of the rotating speed Ω, the following system parameters are always fixed as follows.


$$\zeta =0.05,\beta =0.04,\mu =0.08,R_{0}=1.05$$ (4)

as those from the model in [16,17,41] and the test rig in [10]. Then the non-dimensional governing equation is rewritten as the first-order equation with the initial condition of $\left(X=0,Y=0,X^{\prime }=0,Y^{\prime }=0\right)$, and integrated numerically by fourth-order Runge-Kutta method to obtain the deflection amplitude of the high-speed rotor in the rotor/stator rubbing system. Through the brute-force numerical bifurcation analysis with the variation of the control parameter κ, the optimal value of κ is determined as κ=73.35. With the increase of the rotating speed Ω from 0 to 4, the bifurcation diagram of the rotor in the piecewise smooth rotor/stator rubbing system is shown in Fig 3(a) and that in the smoothening system with κ=73.35 is shown in Fig 3(b).

From the deflection in x direction of the rotor in Fig 3, the bifurcation diagram of the smoothening rotor/stator rubbing system with κ=73.35 depicted in Fig 3(b), is consistent with that of the piecewise smooth system depicted in Fig 3(a). So the validity of the control parameter of κ=73.35 is verified for the rubbing rotors with high speed. From the consistency of the results, it is also concluded that it is an effective method to transform a piecewise smooth system into a smoothening system. In practice, it should be noticed that a rotor is considered to rotate with high speed when Ω≥1 in the rotor/stator rubbing system.

**Fig 4:**
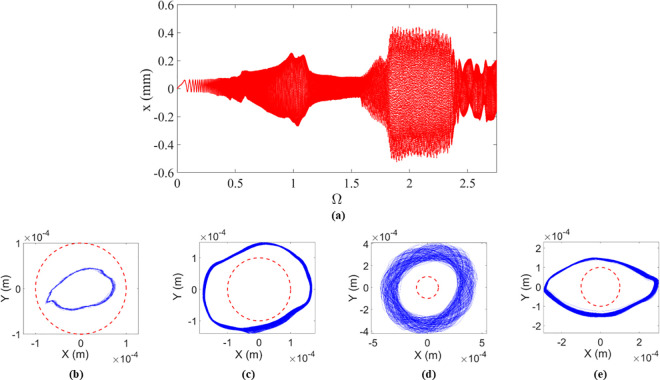
Experimental results of the rotor/stator testing system. **(a) Bifurcation diagram. (b) Orbit with** Ω=0.04. **(c) Orbit with** Ω=1.12. **(d) Orbit with** Ω=2.2. **(e) Orbit with** Ω=2.5**. In Figs 4(b) to 4(e), the clearance between the rotor and the stator is represented by the red dashed cycle with the rotor orbit of blue curves.**

In addition, a machinery fault simulator (MFS) from SpectraQuest^@^, Louisville, USA, is employed to experimentally determine the behaviors of the rotor during the run-up process. The rotor/stator testing system can achieve a maximum speed of 15000 r/min, with a defined clearance of $r_{0}=0.1mm$ between the rotor and the stator. Under indirect measurement of the equivalent stiffness of the shaft, the natural frequency of $\omega_{0}=571rad/s=5457r/min$ of the coupled rotor/stator testing system is obtained. As the rotating speed ω of the rotor progressively increases from 0 to 15000 r/min, i.e., the normalized rotating speed Ω grows from 0 to 2.7, with $c_{s}=139\;Ns/m$, μ=0.12, and e=0.0668 mm, the experimental results of the rotating speed-dependent variable deflection of the rotor in x direction are displayed in Fig 4. In the bifurcation diagram shown in Fig 4(a), the rotor transitions from a periodic no-rub motion to a periodic/quasi-periodic rubbing motion. Considering unavoidable testing errors, the deflection of the rotor in periodic motion fluctuates over a narrow circle, evident in the periodic no-rub motion with Ω=0.04 in Fig 4(b), the periodic synchronous full annular rub with Ω=1.12 in Fig 4(c) and the same type of rub with Ω=2.5 in Fig 4(e). On the contrary, the deflection of the rotor in quasi-periodic motion covers a wider circle area, as shown in the quasi-periodic dry friction backward whirl with Ω=2.2 of Fig 4(d).

As the rotating speed grows with Ω>1, it can be observed from Fig 4 that a ‘jump’ phenomenon between periodic and quasi-periodic motions occurs, marked by the deflection of the high-speed rotor sharply rising from 0.1 mm to 0.4 mm at Ω=1.58. Subsequently, when Ω=2.42, the deflection of the rotor ‘jumps’ back down from 0.4 mm to 0.2 mm. By qualitatively comparing experimental outcomes with the numerical results found in Fig 3, it is apparent that the behaviors of the high-speed rotor in the rotor/stator testing system align with those derived from numerical simulation of the piecewise smooth/smoothening governing equation. Hence the piecewise smooth/smoothening governing equation of the rotor/stator rubbing system with high speed is valid and reasonable for investigating the transition between periodic and quasi-periodic motions.

From the experimental and numerical results of bifurcation diagrams, it is seen that the switching scenario with the increase of rotating speed Ω is something like: periodic motion → quasi-periodic motion → periodic motion. In other words, only the period-one and quasi-periodic attractors appear, which can also be detected through numerical simulation in a micro-rotor system [15], an overcritical high-speed rotor system [39] and a rotor/stator model of a turbogenerator [40]. During the process from periodic motion to quasi-periodic motion or from quasi-periodic motion to periodic motion, the ‘jump’ phenomena appear, in which the ‘jump’ points are defined as bifurcation points and draw enough attention. And whereas the quasi-periodic motion in the smoothening system is triggered by the imbalance with the rotating speed of Ω=1.257, which is lightly smaller than the bifurcation point of Ω=1.280 in the piecewise smooth system. Despite the tiny gap, the bifurcation points of the smoothening rotor/stator rubbing system coincides with those of the piecewise smooth rotor/stator rubbing system. When Ω=2.485, the quasi-periodic motion ceases to exist and period motion is triggered by imbalance again.

As a result of the ‘jump’ phenomena between period and quasi-periodic motions, it is very difficult to capture the numerical proof of the bifurcation and explain the phenomena in the piecewise smooth rotor/stator rubbing system. Therefore, with the aid of the proximate smoothening function of sigm(R), the abundant dynamic behaviors originating from the variation of the system parameters and initial conditions, can be studied by the global analysis of bifurcations in the smoothening rotor/stator rubbing system with the appropriate control parameter of κ=73.35 under high-speed rotating operation.

## 3 Dynamic behaviors of the high-speed rotor/stator rubbing system

**Fig 5:**
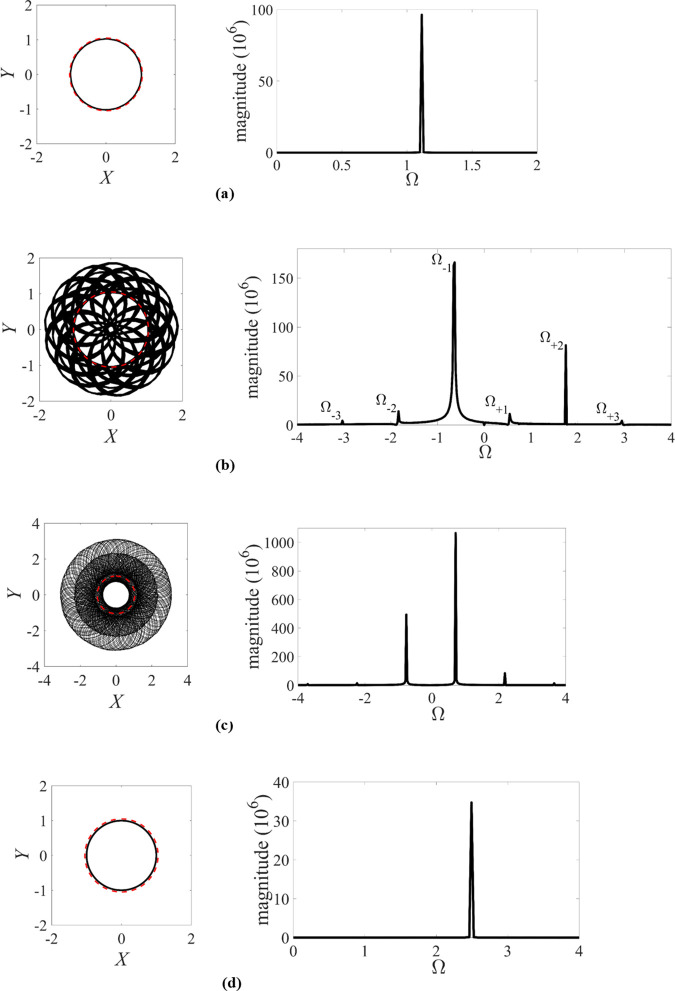
Orbits and full spectra of the rotor with ζ=0.05, β=0.04, μ=0.08, $R_{0}=1.05$ and κ=73.35. (a) Periodic motion with Ω=1.12. (b) Quasi-periodic motion with Ω=1.75. (c) Quasi-periodic motion with Ω=2.1875. (d) Periodic motion with Ω=2.5. In the orbits, the rotor/stator clearance is represented by the red dashed cycle.

The rotor/stator rubbing system with the parameters of ζ=0.05, β=0.04, μ=0.08 and $R_{0}=1.05$, as documented in the literature [10,16,17,41], is examined for the high-speed performance in the work. With the variation of the rotating speed Ω, the global dynamic responses of the rotor/stator rubbing system can be delineated by numerical simulation and theoretical analysis in the smoothening system with the control parameter of κ=73.35. The numerical simulation of the smoothening rotor/stator rubbing system is tackled with the aid of orbit analysis, phase diagrams, Poincaré sections, Lyapunov exponents and full spectra. Based on the bifurcation diagram of the smoothening rotor/stator rubbing system, the orbits and full spectra of the rotors with the high rotating speed of Ω=1.12, Ω=1.75, Ω=2.1875 and Ω=2.5, are respectively depicted in Fig 5.

In Fig 5(a), the period motion occurs with the rotating speed of Ω=1.12, which is smaller than the rotating speed of Ω=1.257 for the onset of the quasi-periodic motion. The orbit of the rotor represented by the solid black lines is less than the clearance represented by the red dashed cycle, i.e., $R<R_{0}$. The full spectra derived from Fast Fourier transform on both variables *X* and *Y*, can yield both positive and negative frequency components respectively representing the forward and backward motion. The response is no-rub motion with the positive frequency of Ω=1.12, due to the forward harmonic excitation.

In Fig 5(b), the quasi-periodic motion occurs with the rotating speed of Ω=1.75. The orbit of the rotor represented by the solid black lines is partly greater than the clearance represented by the red dashed cycle, i.e., $R<R_{0}$ or $R\geq R_{0}$. Moreover, the deflection R of the rotor is somewhat bouncing. From full spectrum, the system response is partial rub with backward whirl frequencies of $\Omega_{-1}$, $\Omega_{-2}$, …, $\Omega_{-n}(n=1,2,...)$ due to friction induced nonlinear modal motion, together with the positive frequencies of $\Omega_{+1}$, $\Omega_{+2}$, …, $\Omega_{+n}(n=1,2,...)$. The second positive frequency of $\Omega_{+2}$ is equal to the frequency Ω of harmonic excitation, i.e., $\Omega_{+2}=\Omega =1.75$. The quasi-periodic responses with multiple positive and negative frequency components during high-speed rotating are vastly different from the quasi-periodic motion with only one exciting frequency and one whirling frequency during low-speed running with Ω<1 in [18,20,21].

In Fig 5(c), the quasi-periodic motion occurs with the rotating speed of Ω=1.1875, which almost has the same partial-rub characteristics with the quasi-periodic motion with Ω=1.75 in Fig 5(b). Similarly, the deflection R of the rotor in Fig 5(c) fluctuates over a wide range with multiple positive and negative frequency components. In Fig 5(d), the period motion occurs with the rotating speed of Ω=2.5, which is bigger than the rotating speed of Ω=2.485 for the cease of the quasi-periodic motion. It is also seen from the orbit and the full spectrum of the rotor in Fig 5(d) that the response shows nearly the same no-rub characteristics with the forward excitation frequency of the high-speed rotor.

Based on the response characteristics of the high-speed rotor, the frequency of period motion and the second frequency of quasi-periodic motion are respectively equal to the frequencies of the harmonic excitation in a rotor/stator rubbing system. According to a plethora of simulation data, the frequency of the whirling rotor in the rotor/stator rubbing system can be obtained as


$$\left\{ \begin{array}{c} \Omega_{+n}=n\Omega_{+1}-(n-1)\Omega_{-1}\\ \Omega_{-n}=n\Omega_{-1}-(n-1)\Omega_{+1} \end{array}\right.(n\in N_{+})$$ (5)

where $N_{+}$ denotes the set of all positive integers.

Taking the rotor/stator rubbing system with Ω=1.75 for example, it is concluded from Fig 5(b) that the frequency values of $\Omega_{+n}$ and $\Omega_{-n}$ satisfy the frequency relation of Eq (5). Then the frequency values of $\Omega_{+n}$ and $\Omega_{-n}$ for partial rub with Ω=1.75 are shown in Table 1.

**Table 1:** $\Omega_{+n}$ and $\Omega_{-n}$ for partial rub with Ω=1.75.

Frequency	$\Omega_{-3}$	$\Omega_{-2}$	$\Omega_{-1}$	$\Omega_{+1}$	$\Omega_{+2}$	$\Omega_{+3}$
**Value**	−3.01	−1.82	−0.63	0.56	1.75	2.94

**Fig 6:**
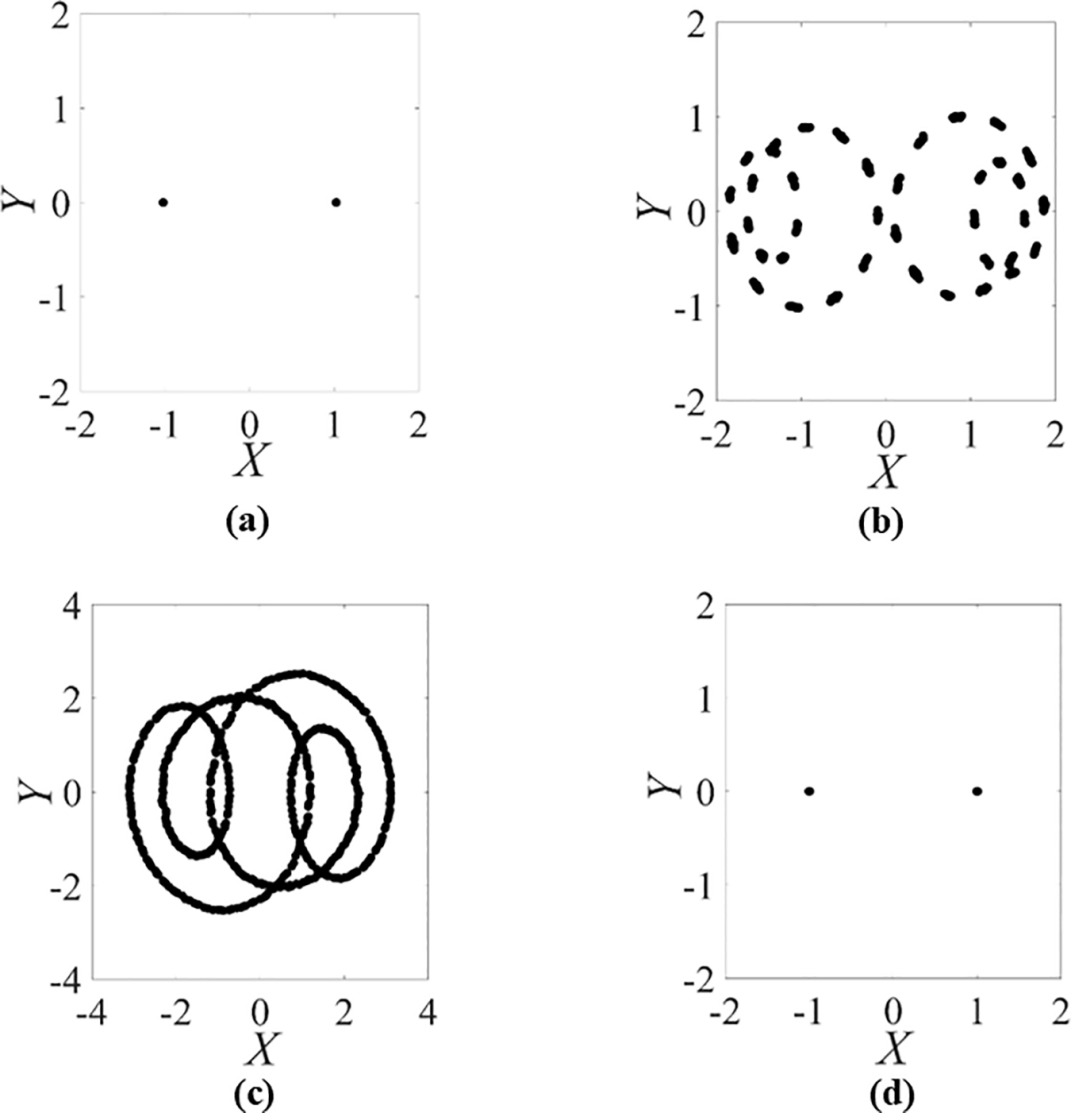
Poincaré sections of the rotor/stator rubbing system with ζ=0.05, β=0.04, μ=0.08, $R_{0}=1.05$ and κ=73.35. (a) Periodic motion with Ω=1.12. (b) Quasi-periodic motion with Ω=1.75. (c) Quasi-periodic motion with Ω=2.1875. (d) Periodic motion with Ω=2.5.

Then the Poincaré sections of the rotor/stator rubbing systems with the different rotating speeds of Ω=1.12, Ω=1.75, Ω=2.1875 and Ω=2.5, are respectively showed in Fig 6. From the projections of Poincaré sections for Ω=1.12 and Ω=2.5 in Figs 6(a) to 6(d), there are two isolated points respectively. Then taking into account only one discrete frequency component in full spectrum, a limited circle in orbit and two isolated points in Poincaré section, it is proved that the motions of the rotor with Ω=1.12 and Ω=2.5 are clearly periodic-one. From the projections of Poincaré sections for Ω=1.75 and Ω=2.1875 in Figs 6(b) and 6(c), there is a closed circle respectively. The trajectory of the rotor is irregular. The corresponding Lyapunov exponent is zero. Then taking into account six discrete frequency components in full spectrum, a limited circle in orbit and a closed circle in Poincaré section, it is proved that the motions of the rotor with Ω=1.75 and Ω=2.1875 are quasi-periodic and the quasi-periodic motion remains from Ω=1.257 to Ω=2.485. So, it is illustrated that just the periodic-one and quasi-periodic attractors appear and the strange attractors do not occur in the rotor/stator rubbing system with high speed.

**Fig 7:**
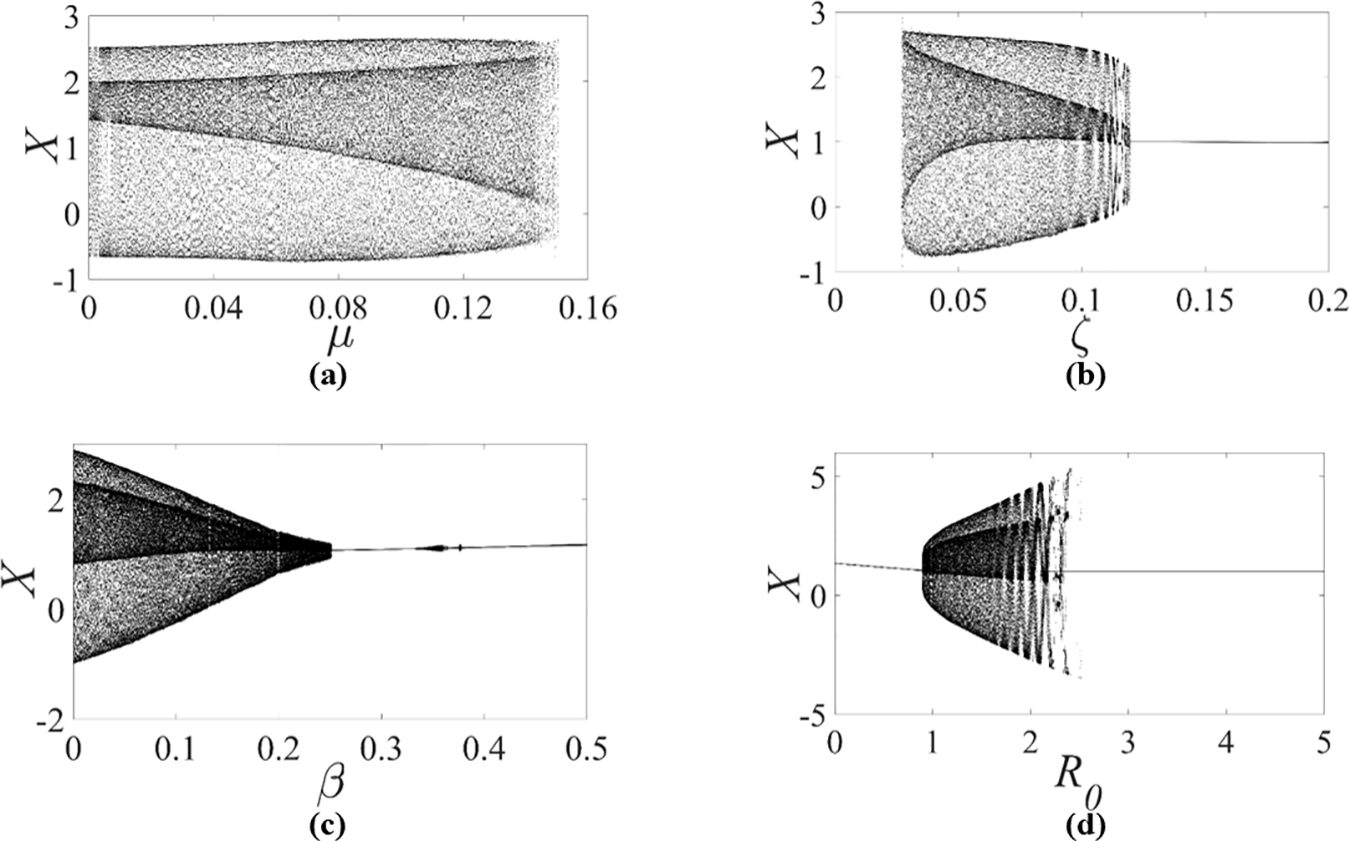
Bifurcation diagrams of the smoothening rotor/stator rubbing system with Ω=2.0 and κ=73.35, during (a) ζ=0.05, β=0.04, $R_{0}=1.05$, (b) β=0.04, μ=0.08, $R_{0}=1.05$, (c) ζ=0.05, μ=0.08, $R_{0}=1.05$, (d) ζ=0.05, β=0.04, μ=0.08.

When Ω=2.0 with the initial condition of $\left(X=0,Y=0,X^{\prime }=0,Y^{\prime }=0\right)$, the dynamic characteristics of the smoothening rotor/stator rubbing system are studied by the aid of the bifurcation diagrams with the variation of system parameters. Fig 7(a) shows the bifurcation diagram with the variation of μ when ζ=0.05, β=0.04 and $R_{0}=1.05$. Fig 7(b) shows the bifurcation diagram with the variation of ζ when β=0.04, μ=0.08 and $R_{0}=1.05$. Fig 7(c) shows the bifurcation diagram with the variation of β when ζ=0.05, μ=0.08 and $R_{0}=1.05$. Fig 7(d) shows the bifurcation diagram with the variation of $R_{0}$ when ζ=0.05, β=0.04 and μ=0.08. It is seen that the chaotic phenomena do not appear in the rotor/stator rubbing system with the variation of μ, ζ, β and $R_{0}$. In addition, it is also concluded from Figs 7(a) and 7(b) that dry friction backward whirl occurs when μ≥0.1504 or ζ≤0.0269, which conforms to the analytical solutions of the existence condition of dry friction backward whirl, i.e., $\mu \geq 2\zeta \sqrt{\beta +1}$ or ζ≤μ/μ(2β+1)\nulldelimiterspace(2β+1), for the rotor/stator rubbing system in full speed range, as illustrated in [17,18]. So, the accuracy of the smoothening model of the rotor/stator rubbing system is confirmed through the consistent results of both numerical simulation and analytical solutions.

## 4 Stability of high-speed rotor responses

According to the results of the numerical simulation in the smoothening rotor/stator rubbing system with high speed, period motion and quasi-periodic motion can dramatically switch as the rotating speed of the rotor increases. It is valuable to reveal the characteristics of the ‘jump’ phenomena from period motion to quasi-periodic motion or from quasi-periodic motion to period motion, which have been analytically studied by the assembling of each local subsystem derived from the discretizing of the solutions in the holonomic system [15,16]. Therefore, the global characteristics of the high-speed rubbing rotors should be elaborated by the smoothening functions with the control parameter of κ=73.35.

### 4.1 Full annular rub solutions of periodic motion

By denoting $\eta =[X,Y,X^{\prime },Y^{\prime }]$, the governing equation of the smoothening rotor/stator rubbing system in Eq (3) can be reformed as a set of first order autonomous ordinary differential equations with smoothening right-hand side.


$$\{\begin{array}{c} {}*20l{\eta_{1}}^{\prime }=\eta_{3}=X^{\prime }\\ {\eta_{2}}^{\prime }=\eta_{4}=Y^{\prime }\\ {\eta_{3}}^{\prime }=-2\zeta \eta_{3}-\beta \eta_{1}-\frac{1}{1+e^{\kappa (R_{0}-R)}}(1-\frac{R_{0}}{R})(X-\mu Y)+\Omega^{2}\cos\Omega \tau \\ {\eta_{4}}^{\prime }=-2\zeta \eta_{4}-\beta \eta_{2}-\frac{1}{1+e^{\kappa (R_{0}-R)}}(1-\frac{R_{0}}{R})(\mu X+Y)+\Omega^{2}sin\Omega \tau \end{array}$$ (6)

According to the orbit, the full spectra and the Poincaré sections of the period motion in Figs 4 and 5, the whirling frequency of the full annular rub response is equal to the rotating speed of the rotor. Then, the reasonable form of the periodic solution of Eq (6) can be determined as


$$\left\{ \begin{array}{c} \eta_{1}=A\;cos(\Omega \tau +\varphi )\\ \eta_{2}=A\;sin(\Omega \tau +\varphi ) \end{array}\right.$$ (7)

where *A* and φ are respectively the amplitude and the phase angle.

Substituting the solution of Eq (7) into Eq (6), it yields


$$\left\{ \begin{array}{c} (\beta -\Omega^{2})A+\frac{\left(A-R_{0}\right)}{1+e^{\kappa (R_{0}-A)}}=\Omega^{2}\cos\varphi \\ 2\zeta \Omega A+\frac{\mu (A-R_{0})}{1+e^{\kappa (R_{0}-A)}}=-\Omega^{2}\sin\varphi \end{array}\right.$$ (8)

Then a polynomial with the amplitude *A* is got as


$$c_{2}A^{2}+c_{1}A+c_{0}=0$$ (9)

where $c_{2}=[\frac{1}{1+e^{\kappa (R_{0}-A)}}+\beta -\Omega^{2}{]}^{2}+[2\zeta \Omega +\frac{\mu }{1+e^{\kappa (R_{0}-A)}}{]}^{2}$,

$c_{1}=\frac{-2R_{0}(1+\mu^{2})}{{\left[1+e^{\kappa (R_{0}-A)}\right]}^{2}}+\frac{-2R_{0}(\beta +2\mu \zeta \Omega -\Omega^{2})}{1+e^{\kappa (R_{0}-A)}}$ and $c_{0}=\frac{R_{0}^{2}(1+\mu^{2})}{{\left[1+e^{\kappa (R_{0}-A)}\right]}^{2}}-\Omega^{4}$.

From the practical point of view, the amplitude of *A* in Eq (7) not only needs to be real and positive but also ought to be greater than the clearance $R_{0}$ between the rotor and the stator. Then the existence condition of *A* is


$$imag(A)=0\;and\;A\geq R_{0}$$ (10)

### 4.2 Stability analysis of periodic solution

By using the theory of matrix characteristic root, the eigenvalues of Jacobian matrix of the linearized governing equation of the rotor/stator rubbing system are introduced and analyzed to study the stability of the periodic solutions. According to the Poincaré sections of the period motion in Fig 5, the equilibrium point $\eta^{0}$ of Eq (6) is defined as


$$\left\{ \begin{array}{c} {\eta_{1}}^{0}=A^{0}\cos(\Omega \tau +\varphi^{0})=A^{0}\cos\theta^{0}\\ {\eta_{2}}^{0}=A^{0}sin(\Omega \tau +\varphi^{0})=A^{0}\sin\theta^{0} \end{array}\right.$$ (11)

where $A^{0}$ is the amplitude, which is achieved by the solution of Eq (7).

From the governing equation of Eq (6), the linearized equation in terms of the equilibrium point of Eq (11) is given as


$$\begin{array}{c} \delta \eta^{\prime }=Df{|}_{\eta =\eta^{0}}\;\delta \eta =\left[J\right]\delta \eta \end{array}$$ (12)

where *D* is derivative operator and [J] is the Jacobian matrix.

The Jacobian matrix [J] of the system is


$$\left[J\right]=\left[\begin{array}{cccc} {}*20c0 & 0 & 1 & 0\\ 0 & 0 & 0 & 1\\ \begin{array}{c} -\beta -B-C\cdot cos^{2}\theta \\ +\mu C\cdot \sin\theta \cos\theta \end{array} & \begin{array}{c} \mu B+\mu C\cdot sin^{2}\theta \\ -C\cdot \sin\theta \cos\theta \end{array} & -2\zeta & 0\\ \begin{array}{c} -\mu B-\mu C\cdot cos^{2}\theta \\ -C\cdot \sin\theta \cos\theta \end{array} & \begin{array}{c} -\beta -B-C\cdot sin^{2}\theta \\ -\mu C\cdot \sin\theta \cos\theta \end{array} & 0 & -2\zeta \end{array}\right]$$ (13)

where $B=\frac{1}{1+e^{\kappa (R_{0}-A^{0})}}(1-\frac{R_{0}}{A^{0}})$ and


$$C=\frac{\kappa \cdot e^{\kappa (R_{0}-A^{0})}}{{\left[1+e^{\kappa (R_{0}-A^{0})}\right]}^{2}}(A^{0}-R_{0})+\frac{1}{1+e^{\kappa (R_{0}-A^{0})}}\frac{R_{0}}{A^{0}}.$$

From Eq (13), the Jacobian matrix [J] is periodic time-dependent, by which the time-dependent eigenvalues cannot be utilized to assess the stability of the rotor/stator rubbing system. Therefore, the coordinate transformation relation of Eq (14) is employed to transform the time-dependent Jacobian matrix [J] to the time-independent Jacobian matrix $\left[J_{0}\right]$.


$$\begin{array}{c} \delta \eta =\left[T\right]\delta U \end{array}$$ (14)

where $\left[T\right]=\left[\begin{array}{cccc} {}*20c\cos\theta & -\sin\theta & 0 & 0\\ \sin\theta & \cos\theta & 0 & 0\\ 0 & 0 & \cos\theta & -\sin\theta \\ 0 & 0 & \sin\theta & \cos\theta \end{array}\right]$.

From $\delta \eta^{\prime }={\left[T\right]}^{\prime }\delta U+\left[T\right]\delta U^{\prime }$, it yields


$$\begin{array}{c} \delta U^{\prime }=\left[J_{0}\right]\delta U \end{array}$$ (15)

where $\left[J_{0}\right]={\left[T\right]}^{-1}(\left[J\right]\left[T\right]-{\left[T\right]}^{\prime })$

Then the time-independent Jacobian matrix $\left[J_{0}\right]$ is derived.


$$\left[J_{0}\right]=\left[\begin{array}{cccc} {}*20c0 & \Omega & 1 & 0\\ -\Omega & 0 & 0 & 1\\ -\beta -B-C & \mu B & -2\zeta & \Omega \\ -\mu (B+C) & -\beta -B & -\Omega & -2\zeta \end{array}\right]$$ (16)

With the time-independent Jacobian matrix $\left[J_{0}\right]$ of Eq (16), the real parts of its eigenvalues can be used for the stability criterion of δU, i.e., δη and $\eta^{0}$, and thus the stability of the full annular rub in the rotor/stator rubbing system. When all the real parts of the eigenvalues of $\left[J_{0}\right]$ are negative, the system is defined as stability, but not vice versa.

From $\left|J_{0}-\lambda I\right|=0$ with Ω, the characteristic equation of $\left[J_{0}\right]$ in terms of the eigenvalues λ can be determined as the following polynomial equation.


$$\lambda^{4}+4\zeta \lambda^{3}+b_{2}\lambda^{2}+b_{1}\lambda +b_{0}=0$$ (17)

where λ represents the eigenvalues of the Jacobian matrix $\left[J_{0}\right]$ and


$$b_{2}=4\zeta^{2}+2(\beta +B)+2\Omega^{2},$$

$$b_{1}=4\zeta (\beta +B)+2(2\mu B+C)\Omega +4\zeta \Omega^{2},$$

$$\begin{array}{c} b_{0}=\Omega^{4}+(4\zeta^{2}-C)\Omega^{2}+2[\mu \zeta (B+C)-2(\beta +B)Omega\\ \;+(\beta +B)(\beta +B+C)+\mu^{2}B(B+C) \end{array}$$

Then the two pairs of the eigenvalues, i.e., $\lambda_{1}$, $\lambda_{2}$ and $\lambda_{3}$, $\lambda_{4}$, can be obtained by the solutions of Eq (17), which respectively vary with the rotating speed Ω of the rotor in the rotor/stator rubbing system.

**Fig 8:**
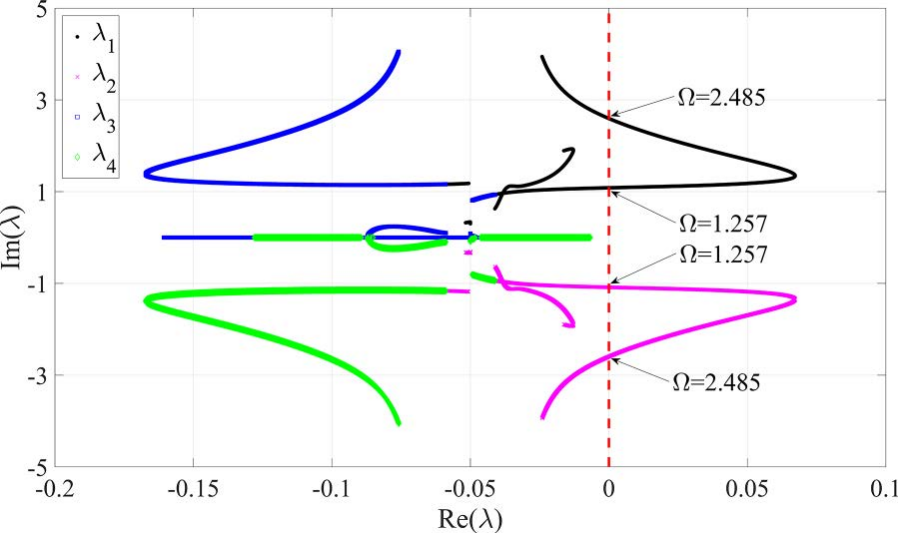
Behaviors of eigenvalues with the variation of Ω from 0 to 4 with ΔΩ=0.0001 in the smoothening rotor/stator rubbing system with ζ=0.05, β=0.04, μ=0.08, $R_{0}=1.05$ and κ=73.35. **The red dashed line represented the stability boundary with** Re\nolimits(λ)=0.

According to the dynamic behaviors of the high-speed rotor/stator rubbing system, only the period motion and quasi-periodic motion can occur. Then the switch between the period motion and the quasi-periodic motion can be detected by the transition between the stable and unstable states of the periodic solutions. Thus the alternation of the real parts of $\lambda_{1}$, $\lambda_{2}$ and $\lambda_{3}$, $\lambda_{4}$ from positive to negative, or from negative to positive, can decide the ‘jump’ phenomena of the period motion and the quasi-periodic motion in the rotor/stator rubbing system with high speed. As the rotating speed Ω varies between 0 and 4 with the step size of ΔΩ=0.0001, the behaviors of the real parts Re\nolimits(λ) and the imaginary parts Im\nolimits(λ) of the eigenvalues $\lambda_{1}$, $\lambda_{2}$ and $\lambda_{3}$, $\lambda_{4}$ are obtained, as shown in Fig 8.

From Fig 8, it is seen that the real parts of eigenvalues $\lambda_{3}$ represented by blue squares and $\lambda_{4}$ represented by green diamonds are always negative with positive or negative imaginary parts during the variation of Ω∈[0,4]. While the real parts of eigenvalues $\lambda_{1}$ represented by black dots and $\lambda_{2}$ represented by magenta crosses can alter between the positive and the negative. Therefore, the stability of the rotor/stator rubbing system can be decided by the negative real parts of the eigenvalues $\lambda_{1}$ and $\lambda_{2}$. It is concluded that the real parts of $\lambda_{1}$ and $\lambda_{2}$ crosses over the critical points with Re\nolimits(λ)=0, namely stability boundary represented by the red dashed line, when Ω=1.257 and Ω=2.485. The critical rotating speeds are also deemed as the bifurcation points which coincides with the ‘jump’ points from period motion to quasi-periodic motion and from quasi-periodic motion to period motion in Figs 3 and 4.

According to the characteristics of bifurcation points and the distribution of the two pairs of the eigenvalues $\lambda_{1}$, $\lambda_{2}$ and $\lambda_{3}$, $\lambda_{4}$, the stability of the periodic solutions can also be ascertained by the algebraic criterion of Saddle-node bifurcation and Hopf bifurcation in the smoothening rotor/stator rubbing system with high speed. Therefore, the occurrence from period motion to quasi-periodic motion or from quasi-periodic motion to period motion can be elaborately explained from the point of view of bifurcation theory.

When one of the eigenvalues $\lambda_{1}$, $\lambda_{2}$ and $\lambda_{3}$, $\lambda_{4}$ of the Jacobin matrix $\left[J_{0}\right]$ is equal to zero, saddle-node bifurcation occurs in the rotor/stator rubbing system. From Eq (17) with the existence of a zero eigenvalue, the saddle-node bifurcation condition is derived by $b_{0}=0$. That is


$$\begin{array}{c} \Omega^{4}+(4\zeta^{2}-C)\Omega^{2}+2[\mu \zeta (B+C)-2(\beta +B)Omega\\ +(\beta +B)(\beta +B+C)+\mu^{2}B(B+C)=0 \end{array}$$ (18)

The positive solutions of Ω can be used to determine the existence boundary of saddle-node bifurcation in the parameter space of the rotor/stator rubbing system.

When the eigenvalues $\lambda_{1}$, $\lambda_{2}$ and $\lambda_{3}$, $\lambda_{4}$ are conjugated complex numbers wherein a pair of pure imaginary values are given in the Jacobin matrix $\left[J_{0}\right]$, Hopf bifurcation appears in the rotor/stator rubbing system. Then the eigenvalues $\lambda_{1}$, $\lambda_{2}$ and $\lambda_{3}$, $\lambda_{4}$ can be denoted as


$${\bar{\lambda }}_{1,2}=\pm i\varpi ,\;{\bar{\lambda }}_{3,4}=c\pm id$$ (19)

where $i=\sqrt{-1}$.

Taking ${\bar{\lambda }}_{1}$, ${\bar{\lambda }}_{2}$, ${\bar{\lambda }}_{3}$ and ${\bar{\lambda }}_{4}$ as the solutions of a polynomial equation, the characteristic equation of the rotor/stator rubbing system can be given as the following equation.


$$\lambda^{4}-2c\lambda^{3}+(c^{2}+d^{2}+\varpi^{2})\lambda^{2}-2c\varpi^{2}\lambda +\varpi^{2}(c^{2}+d^{2})$$ (20)

Through comparison between Eq (17) and Eq (20), it yields


$$4\zeta =-2c,b_{2}=c^{2}+d^{2}+\varpi^{2},b_{1}=-2c\varpi^{2},b_{0}=\varpi^{2}(c^{2}+d^{2})$$ (21)

From Eq (21), the equation of $b_{0}$, $b_{1}$ and $b_{2}$ is obtained.


$$16\zeta^{2}b_{0}=4\zeta b_{1}b_{2}-b_{1}^{2}$$ (22)

Substituting the mathematic representations of $b_{0}$, $b_{1}$ and $b_{2}$ in Eq (17) into Eq (22), the equation in terms of Ω is obtained and solved for the critical rotating speed Ω, i.e., the existence boundary of Hopf bifurcation in the rotor/stator rubbing system.

For the smoothening rotor/stator rubbing system with ζ=0.05, β=0.04, μ=0.08, $R_{0}=1.05$ and κ=73.35, the critical rotating speed Ω of Hopf bifurcation is theoretically solved as Ω=1.257 or Ω=2.485, which is in accordance with the numerical results derived by numerical simulation and stability analysis. It is elaborated from the results in a good agreement that the ‘jump’ phenomena between period motion and quasi-periodic motion are all ascribed to Hopf bifurcation in the rotor/stator rubbing system with high speed.

## 5 Influences of system parameters on high-speed rotor responses

From the discussion in the bifurcation of the smoothening rotor/stator rubbing system, it is noted that the dynamic behaviors can be influenced by the system parameters. With the aid of a bifurcation analysis tool of MATCONT [43], which is mainly used for the continuous system and/or the autonomous system, the characteristics of the system responses are illustrated versus the rotating speed Ω with the variation of the control parameter κ and friction coefficient μ, through brute-force numerical bifurcation analysis.

### 5.1 Influence of control parameter κ

**Fig 9:**
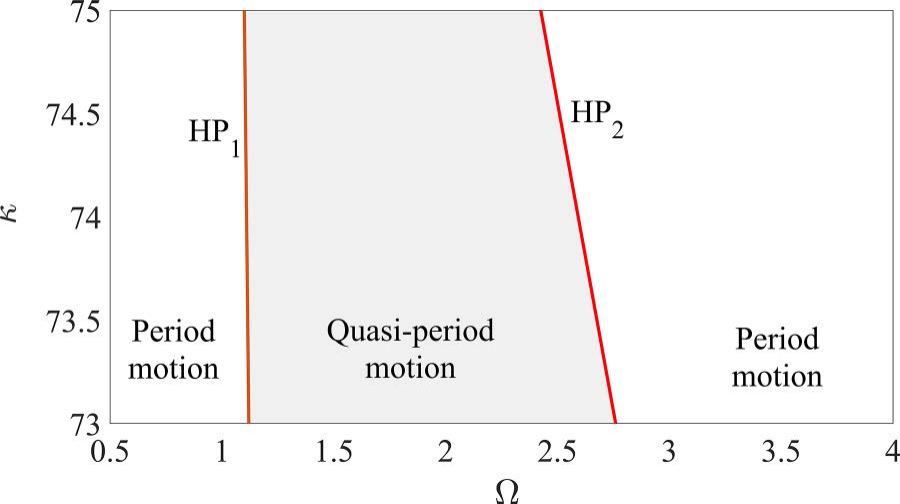
Plot of κ−Ω when ζ=0.05, β=0.04, μ=0.08 and $R_{0}=1.05$. Lines HP_**1**_ and HP_**2**_ are the Hopf bifurcation boundaries of periodic motion.

By fixing ζ=0.05, β=0.04, μ=0.08 and $R_{0}=1.05$ with κ∈[73,75] and different Ω, the plot of control parameter versus rotating speed, namely on the parameter plane κ−Ω, is shown in Fig 9. Curves HP_1_ and HP_2_ indicate the rotating speed whereby the ‘jump’ phenomena between period motion and quasi-periodic motion occur due to Hopf bifurcation. It is interesting to note that the rotating speed Ω of curve HP_1_ varies from 1.12 to 1.10 while Ω of curve HP_2_ varies from 1.76 to 2.425 when κ∈[73,75]. Therefore, the responses of the whirling rotor primarily depend on the value of the control parameter κ in the smoothening rotor/stator rubbing system. However, the value of the control parameter κ cannot affect the ‘jump’ behavior of the responses of the high-speed rubbing rotor. Considering synthetically the solving precision and the computational expense, the value of the control parameter κ in the smoothening system is not the larger the better, which can be determined by the approximately comparation of the solutions between the smoothening system and the discontinuous system.

### 5.2 Influence of friction coefficient μ

**Fig 10:**
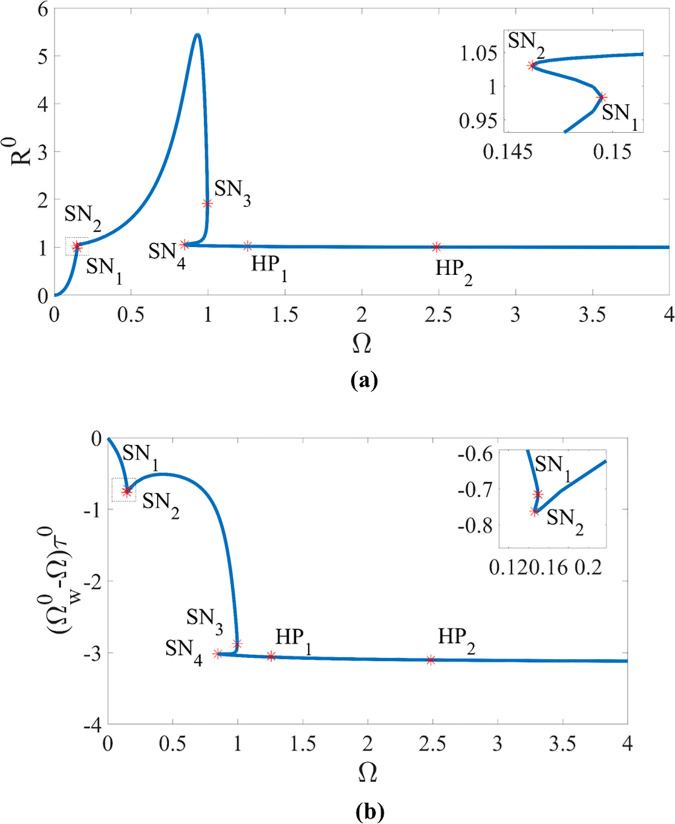
Bifurcation characteristics under different rotating speed of the rotor in the rotor/stator rubbing system with ζ=0.05, β=0.04, μ=0.08, $R_{0}=1.05$ and κ=73.35. (a) $R^{0}$ versus Ω. (b) $({\Omega_{w}}^{0}-\Omega )\tau^{0}$ versus Ω. HP_**1**_ and HP_**2**_ represent the Hopf bifurcation boundaries. SN_**1**_, SN_**2**_, SN_**3**_ and SN_**4**_ represent the saddle-node bifurcation boundaries.

By fixing ζ=0.05, β=0.04, μ=0.08, $R_{0}=1.05$ and κ=73.35, the equilibrium point of $\eta^{0}$ at time $\tau^{0}$ under different rotating speed Ω of the rotor, can be obtained by MATCONT in the rotor/stator rubbing system, as shown in Fig 10. As the rotating speed Ω increases from 0 to 4, the equilibrium curve of the deflection $R^{0}$ that is defined as $R^{0}=\sqrt{{\eta_{1}}^{0}+{\eta_{2}}^{0}}$, is shown in Fig 10(a), while the equilibrium curve of the phase difference $({\Omega_{w}}^{0}-\Omega )\tau^{0}$ where ${\Omega_{w}}^{0}$ is the whirling angular speed of the rotor at time $\tau^{0}$, is shown in Fig 10(b). Points HP_1_ and HP_2_ represent the Hopf bifurcation boundaries, and points SN_1_, SN_2_, SN_3_ and SN_4_ represent the saddle-node bifurcation boundaries. From the existence condition of bifurcation points in Fig 10, the equilibrium solutions SN_1_ of saddle-node bifurcation is (0.9833, −0.7156, 0, 0) at Ω=0.1495 with SN_2_ (1.0305, −0.7628, 0, 0) at Ω=0.1462, SN_3_ (1.9161, −2.8761, 0, 0) at Ω=0.9956 and SN_4_ (1.0555, −3.0164, 0, 0) at Ω=0.8478. In addition, the equilibrium solutions HP_1_ of Hopf bifurcation is (1.0258, −3.0500, 0, 0) at Ω=1.2568 with HP_2_ (1.0046, −3.1043, 0, 0) at Ω=2.4847, which are the supercritical Hopf bifurcation points in the sense of the state evolution from a fixed point to the periodic one.

**Fig 11:**
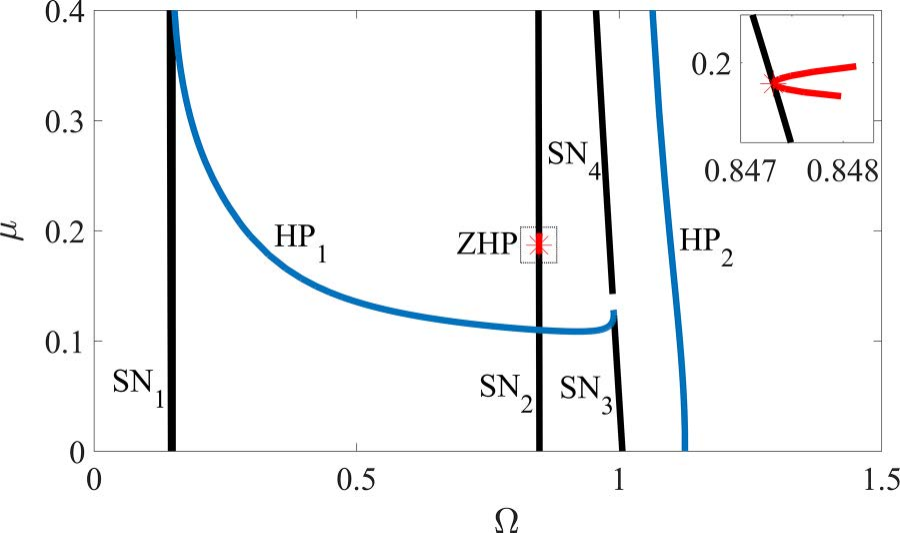
Rotor response characteristics on the plane of Ω−μ, where ζ=0.05, β=0.04, $R_{0}=1.05$ and κ=73.35. Curves HP1 and HP_2_ indicate the rotating speed where the ‘jump’ phenomena between periodic motion and quasi-periodic motion occur. Lines SN_**1**_, SN_**2**_, SN_**3**_ and SN_**4**_ represent the saddle-node bifurcation boundaries. ZHP is Zero-Hopf bifurcation point.

By fixing ζ=0.05, β=0.04, $R_{0}=1.05$ and κ=73.35 with the variation of μ∈[0,0.4] and Ω∈[0,1.5], the global response characteristics on the parameter planes of Ω−μ are depicted as shown in Fig 11. Lines SN_1_, SN_2_, SN_3_ and SN_4_ represent the saddle-node bifurcation boundaries, and curves HP_1_ and HP_2_ represent the Hopf bifurcation boundaries where the ‘jump’ phenomena between periodic motion and quasi-periodic motion occur. The characteristics of the supercritical Hopf bifurcation curve HP_1_ between the two Saddle-node bifurcation curves SN_1_ and SN_2_ are in accordance with those in [8,15,41]. When μ=0.1869 and Ω=0.8473, one of the eigenvalues of the Jacobin matrix is equal to zero, one pair is formed of conjugated imaginary eigenvalues, and the other one is complex number with a non-zero real part. This means a zero-Hopf bifurcation that is also known as a fold-Hopf bifurcation represented by the point ZHP in Fig 11 appears. From the isolated zero-Hopf equilibrium point, the rotor/stator rubbing system undergoes a change in behavior, and a local chaos may birth under certain conditions, which has been detected by numerical simulation in [15,39]. According to the comparison between the numerical simulation results and the theoretical bifurcation boundaries, the agreements of the global dynamic characteristics indicate the ability of the smoothening system based on a sigmoid function sigm(R) in dealing with the bifurcation behaviors of the rotor/stator rubbing system with high speed.

From above discussion, the smoothening sigmoid function is employed in the rotor/stator rubbing system to highlight its benefits and explore the global dynamic characteristics, such as detecting the onset of the rotor responses, identifying the boundaries between periodic and quasi-periodic motions via stability analysis and bifurcation theory, and assessing the influences of the system parameters on design. Results from numerical simulation and theoretical analysis reveal that not only the global responses but also their corresponding evolution can be accurately captured by the proposed rotor/stator system with smoothening function. Moreover, the application of smoothening function to the global dynamic analysis of the rotor/stator rubbing system embarks upon the holonomic solutions rather than the discrete solutions in [15]. Consequently, the smoothening function offers more benefits compared to Heaviside function. Nevertheless, caution should be taken while selecting the control parameter κ of the smoothening function, as it can significantly influence the global response characteristics, posing challenges in achieving the reasonable tradeoff between accuracy and cost. Additionally, adopting the proposed smoothening method allows for addressing more intricate and detailed issues of the rubbing rotors efficiently, ensuring smooth operation of the rotor during rubbing without the failure under any circumstances.

## 6 Conclusions

In this paper, the global dynamic characteristics of a piecewise smooth rotor/stator rubbing system with high speed are presented through analytical analysis and numerical simulation. A method is introduced to determine the global response characteristics of the smoothening system, which involves analyzing the smoothening equation of motion by replacing the Heaviside function with the sigmoid function. By comparing the dynamic behaviors of the piecewise smooth system and smoothening system, the control parameter of smoothness is determined in the sense of the tradeoff between the computational cost and the accuracy of global responses. Finally, the study integrates periodic and quasi-periodic motions within the same parameter space to derive the global response characteristics of the smoothening rotor/stator rubbing system through numerical simulation and stability analysis.

From the point view of global responses, the switching scenario of the rotor/stator rubbing system with high speed follows: periodic motion → quasi-periodic motion → periodic motion, indicating the absence of chaotic behavior. Bifurcation diagrams align well with numerical orbits and Poincaré sections of periodic-one and quasi-periodic attractors. During high-speed rotating, the frequencies of the whirling rotor align with Eq (5), differing from the analytical solutions in [18,20,21]. Through stability analysis of periodic solutions in high-speed rotor responses, the Hopf bifurcation boundaries identifying ‘jump’ phenomena between periodic and quasi-periodic motions, as well as the saddle-node bifurcation boundaries, are verified. With the aid of the evolution of the equilibrium solutions of Hopf bifurcation and saddle-node bifurcation, the global dynamic characteristics in the parameter planes of rotating speed and dry friction coefficient are obtained, wherein zero-Hopf bifurcation is detected in the rotor/stator rubbing system with high speed. It is observed from the influences of control parameter and friction coefficient that small friction on the contact surfaces can benefit the rotor rubbing behavior by avoiding the occurrence of quasi-periodic motion. The results discussed in this paper provide deep insights into the interactive effect of different parameters on the response characteristics of the high-speed rubbing rotors, consistent with analytical predictions. Furthermore, experimental studies are crucial for validating global behavior, paving the way for future research in the rotor/stator rubbing system with high speed.

### Nomenclature

**Table:** 

$R_{0}$	Non-dimensional clearance
*R*	Non-dimensional deflection of the shaft center
$R_{d}$	Non-dimensional radius of the rotor
*X*, *Y*	Non-dimensional deflections of the shaft center
$c_{s}$	Damping of the rotor, N·s/m
*e*	Rotor mass eccentricity, m
$k_{s}$, $k_{b}$	Stiffness of the rotor shaft and the stator, N/m
*m*	Imbalanced mass of the rotor, kg
$r_{0}$	Clearance between rotor and stator, m
*r*	Defection of the shaft geometric center, m
$r_{d}$	Radius of the disk at contact point, m
*t*	Time, s
β	Stiffness ratio of rotor-to-stator, or contact stiffness ratio, ks/kskb\nulldelimiterspacekb
λ	Eigenvalues of the Jacobian matrix
ϕ	Whirling angel at contact point, rad
μ	Coefficient of friction
τ	Non-dimensional time
κ	Control parameter of smoothness
ω	Rotating speed of the rotor, rad/s
$\omega_{0}$	Natural frequency of the rotor system with zero clearance, rad/s
$\omega_{w}$	Whirling speed of the rotor, rad/s
Ω	Normalized rotating speed of the rotor, ω/ωω0\nulldelimiterspaceω0
$\Omega_{w}$	Normalized whirling speed of the rotor, ωw/ωwω0\nulldelimiterspaceω0
ζ	Damping ratio of the rotor system

## References

[1] Yuan J, Gastaldi C, Denimal Goy E, Chouvion B. Friction damping for turbomachinery: A comprehensive review of modelling, design strategies, and testing capabilities. Progress in Aerospace Sciences. 2024;147:101018. doi: 10.1016/j.paerosci.2024.101018

[2] Tang T, Wang Y, Wang S, Zhang M, Chen Z, Zhao Y. Investigation on dynamic rubbing characteristics of a bladed rotor system with multi-mode rubbing fault. Journal of Sound and Vibration. 2025;596:118790. doi: 10.1016/j.jsv.2024.118790

[3] Ding Q, Feng Z, Zhang Y, Sun W. Dynamic analysis of slant cracked rotor system considering nonlinear oil film force. PLoS One. 2024;19(1):e0294293. doi: 10.1371/journal.pone.0294293 38271385 PMC10810477

[4] Kim YB, Noah ST. Bifurcation analysis for a modified Jeffcott rotor with bearing clearances. Nonlinear Dyn. 1990;1(3):221–41. doi: 10.1007/bf01858295

[5] Ishida Y, Yamamoto T. Forced oscillations of a rotating shaft with nonlinear spring characteristics and internal damping (1/2 order subharmonic oscillations and entrainment). Nonlinear Dyn. 1993;4(5):413–31. doi: 10.1007/bf00053689

[6] Chu F, Zhang Z. Bifurcation and chaos in a rub-impact jeffcott rotor system. Journal of Sound and Vibration. 1998;210(1):1–18. doi: 10.1006/jsvi.1997.1283

[7] Edwards S, Lees AW, Friswell MI. The influence of torsion on rotor/stator contact in rotating machinery. Journal of Sound and Vibration. 1999;225(4):767–78. doi: 10.1006/jsvi.1999.2302

[8] Lu W, Chu F. Radial and torsional vibration characteristics of a rub rotor. Nonlinear Dyn. 2014;76(1):529–49. doi: 10.1007/s11071-013-1147-6

[9] Z C F, X Z Z. Rubbing phenomena in rotor-stator contact. Chaos, Solitons and Fractals. 2002;14:257–67.

[10] Johnson DC. Synchronous Whirl of a Vertical Shaft Having Clearance in One Bearing. Journal of Mechanical Engineering Science. 1962;4(1):85–93. doi: 10.1243/jmes_jour_1962_004_012_02

[11] Billett RA. Shaft whirl induced by dry friction. Engineer. 1965;29:713–4.

[12] Black HF. Interaction of a Whirling Rotor with a Vibrating Stator across a Clearance Annulus. Journal of Mechanical Engineering Science. 1968;10(1):1–12. doi: 10.1243/jmes_jour_1968_010_003_02

[13] Prabith K, Praveen Krishna IR. The numerical modeling of rotor-stator rubbing in rotating machinery: a comprehensive review. Nonlinear Dynamics. 2020;101:1317–63.

[14] Xu X, Han Q, Chu F. Review of Electromagnetic Vibration in Electrical Machines. Energies. 2018;11(7):1779. doi: 10.3390/en11071779

[15] Zhang W-M, Meng G. Stability, bifurcation and chaos of a high-speed rub-impact rotor system in MEMS. Sensors and Actuators A: Physical. 2006;127(1):163–78. doi: 10.1016/j.sna.2005.11.014

[16] Jiang J, Ulbrich H. Stability analysis of sliding whirl in a nonlinear Jeffcott rotor with cross-coupling stiffness coefficients. Nonlinear Dynamics. 2001;24:269–83.

[17] Jiang J, Ulbrich H. The Physical Reason and the Analytical Condition for the Onset of Dry Whip in Rotor-to-Stator Contact Systems. Journal of Vibration and Acoustics. 2004;127(6):594–603. doi: 10.1115/1.1888592

[18] Jiang J. The Analytical Solution and The Existence Condition of Dry Friction Backward Whirl in Rotor-to-Stator Contact Systems. Journal of Vibration and Acoustics. 2006;129(2):260–4. doi: 10.1115/1.2345677

[19] Jiang J. Determination of the global responses characteristics of a piecewise smooth dynamical system with contact. Nonlinear Dynamics. 2009; 57: 351–61.

[20] Chen Y, Jiang J. Determination of nonlinear normal modes of a planar nonlinear system with a constraint condition. Journal of Sound and Vibration. 2013;332(20):5151–61. doi: 10.1016/j.jsv.2013.04.040

[21] Kuether RJ, Steyer A. Large-scale harmonic balance simulations with Krylov subspace and preconditioner recycling. Nonlinear Dyn. 2024;112(5):3377–98. doi: 10.1007/s11071-023-09171-6

[22] Peletan L, Baguet S, Torkhani M, Jacquet-Richardet G. Quasi-periodic harmonic balance method for rubbing self-induced vibrations in rotor–stator dynamics. Nonlinear Dyn. 2014;78(4):2501–15. doi: 10.1007/s11071-014-1606-8

[23] Lingener A. Investigation of reverse whirl of a flexible rotor. Rakenteiden Mekaniikka. 1991;24:3–21.

[24] Muszynska A. Rotordynamics. New York: Taylor & Francis; 2005.

[25] Ishida Y, Yamamoto T. Linear and nonlinear rotordynamics. Weinheim: Wiley-VCH; 2012.

[26] Bently DE, Yu JJ, Goldman P, Muszynska A. Full Annular RUB in Mechanical Seals, Part I: Experimental Results. International Journal of Rotating Machinery. 2002;8(5):319–28. doi: 10.1155/s1023621x02000301

[27] Crandall S. From whirl to whip in rotordynamics. In: Proceedings of the 3rd IFToMM International Conference on Rotordynamics. 1990. p. 19–26.

[28] Prabith K, Praveen Krishna IR. The numerical modeling of rotor–stator rubbing in rotating machinery: a comprehensive review. Journal of Sound and Vibration. 2020;101:1317–63.

[29] Arafa AA, Hamdallah SAA, Tang S, Xu Y, Mahmoud GM. Dynamics analysis of a Filippov pest control model with time delay. Communications in Nonlinear Science and Numerical Simulation. 2021;101:105865. doi: 10.1016/j.cnsns.2021.105865

[30] Leine RI, Nijmeijer H. Dynamics and bifurcations of non-smooth mechanical systems. Springer-Verlag Berlin Heidelberg GmbH; 2004.

[31] Wang S, Hong L, Jiang J. Characteristics of stick-slip oscillations in dry friction backward whirl of piecewise smooth rotor/stator rubbing systems. Mechanical Systems and Signal Processing. 2020;135:106387. doi: 10.1016/j.ymssp.2019.106387

[32] Wang S, Hong L, Jiang J. Nonsmooth Behavior of Sliding Bifurcations in a General Piecewise Smooth Rotor/Stator Rubbing System. Int J Bifurcation Chaos. 2021;31(02):2150085. doi: 10.1142/s0218127421500851

[33] Hosham HA, Alharthi TN. Bifurcation and chaos in simple discontinuous systems separated by a hypersurface. MATH. 2024;9(7):17025–38. doi: 10.3934/math.2024826

[34] Wang S, Hong L, Jiang J. Evaluation on spectral submanifold based reduced models of a rotor/stator rubbing system with cross-coupling stiffness. International Journal of Mechanical Sciences. 2022;228:107486. doi: 10.1016/j.ijmecsci.2022.107486

[35] Fan S, Hong L, Jiang J. Model reduction of high-dimensional self-excited nonlinear systems using floquet theory based parameterization method. Nonlinear Dyn. 2024;113(2):1137–61. doi: 10.1007/s11071-024-10307-5

[36] Wiercigroch M, Kraker BD. Applied nonlinear dynamics chaos of mechanical systems with discontinuous. World Scientific; 2000.

[37] Bernardo M, Budd C, Champneys AR, Kowalczyk P. Piecewise-smooth dynamical systems: theory and applications. Springer Science & Business Media; 2008.

[38] Sun Z, Xu J, Zhou T. Analysis on complicated characteristics of a high-speed rotor system with rub-impact. Mechanism and Machine Theory. 2002;37(7):659–72. doi: 10.1016/s0094-114x(02)00010-1

[39] Grāpis O, Tamužs V, Ohlson N-G, Andersons J. Overcritical high-speed rotor systems, full annular rub and accident. Journal of Sound and Vibration. 2006;290(3–5):910–27. doi: 10.1016/j.jsv.2005.04.031

[40] Roques S, Legrand M, Cartraud P, Stoisser C, Pierre C. Modeling of a rotor speed transient response with radial rubbing. Journal of Sound and Vibration. 2010;329(5):527–46. doi: 10.1016/j.jsv.2009.09.016

[41] Jiang J, Gao WH. Study on responses characteristics of a class of piecewise smooth nonlinear planar motion systems. Chinese Journal of Theoretical and Applied Mechanics. 2013;45(1):16–24.

[42] Srivastava AK, Tiwari M, Singh A. Identification of rotor-stator rub and dependence of dry whip boundary on rotor parameters. Mechanical Systems and Signal Processing. 2021;159:107845. doi: 10.1016/j.ymssp.2021.107845

[43] Govaerts W, Kuznetsov YA, Witte VD, Dhooge A, Meijer HGE, Mestrom W, et al. Matcont and CL-Matcont: Continuation Toolboxes in Matlab. Gent University and Utrecht University; 2011.

